# Current Role of Chemotherapy in Nonmetastatic Nasopharyngeal Cancer

**DOI:** 10.1155/2018/3725837

**Published:** 2018-10-01

**Authors:** Tapesh Bhattacharyya, Geethu Babu, Cessal Thommachan Kainickal

**Affiliations:** ^1^Division of Radiation Oncology, Tata Medical Center, Kolkata, India; ^2^Division of Radiation Oncology, Regional Cancer Centre, Trivandrum, India

## Abstract

Nasopharyngeal carcinoma is highly radio- and chemosensitive tumor with its unique clinical and biological behavior. Treatment of stage I disease is radical radiotherapy alone. For stage II disease treatment is radiotherapy with or without chemotherapy. The standard of care for locally advanced nasopharyngeal cancer (stages III-IVB) is concurrent chemoradiation. Optimum timing and sequence of chemotherapy are not yet well-defined. The role of adjuvant and induction chemotherapy is debatable. Here we are going to highlight the role of chemotherapy in nasopharyngeal carcinoma, its benefit, and controversies regarding timing and sequences.

## 1. Introduction

Carcinoma nasopharynx is a distinct clinical and biological entity as compared to other head and neck squamous cell carcinoma [1]. It is endemic in Southern China, Southeast Asia, Middle East, Alaska, and Greenland. In these areas Epstein Barr virus (EBV) is strongly associated with nasopharyngeal carcinoma (NPC). Most of the patients have nonkeratinizing or poorly differentiated (WHO type II/III) carcinoma. Extensive, disproportionate nodal involvement compared to primary and bilateral nodal involvement is characteristic of NPC [2].

Carcinoma nasopharynx is a highly radiosensitive tumor. Radiotherapy (RT) is the backbone of treatment of NPC. With RT alone 5-year overall survival (OS) rate in early stage NPC is around 90%. However, around 70% of patients present with locally advanced stage and 5-year survival with RT alone are poor [3]. This prompted investigators to add chemotherapy to enhance the effect of radiation in locally advanced nasopharyngeal cancer.

## 2. Role of Radiotherapy Alone in Early Nasopharyngeal Cancer

The treatment of stage I carcinoma nasopharynx is RT alone. Hong Kong group reported 5-year local control and OS rate of 91% and 90%, respectively, for stage I disease treated to conventional RT predominantly [4]. RTOG 0225 trial also showed that none of the patients with early stage disease treated with IMRT alone developed loco-regional failure [5].

The prognosis of patients with stage I disease is extremely good with RT alone but in stage II disease, the outcome is not that impressive. Chua et al. [6] showed that when patients were staged according to 1997 AJCC Classification, patients with stage II disease had a poorer outcome as compared to stage I patients (10-year disease free survival (DFS) 60% versus 98%). Among stage II patients T2N1 stage did worse. Radiotherapy alone may not be the adequate treatment for stage II disease and combined modality treatment is warranted. Xiao et al. [7] also identified Chinese 1992 stage T2N1 as a unique subgroup in early stage NPC with 5-year OS of only 73.1%. Leung et al. [8] showed that isolated distant metastasis occurred in only 5.7% of patients with stage IIA but were found in 14.9% of patients with stage IIB disease. Chen et al. [9] in their phase III trial randomly assigned Chinese 1992 stage II NPC patients to receive either RT alone or chemoradiation (CCRT) (concurrent Cisplatin-30 mg/m2 weekly). At a median follow-up of 5 years the OS significantly improved in the CCRT arm (94.5% versus 85.8%; p=0.007). This was due to improvement in distant metastasis free survival (94.8% versus 83.9%; p=0.007); however, there was no statistically significant difference in loco-regional control. But in the recent era of IMRT, whether radiotherapy alone is sufficient for stage II NPC is debatable. A meta-analysis by Liu et al. [10] reported IMRT alone is comparable to chemoradiation in terms of OS, loco-regional relapse-free survival (LRRFS), and distant metastasis free survival (DMFS) for patients with stage II NPC. But clinical stage II NPC consisted of three subgroups, T2N0M0, T1N1M0, and T2N1M0, with different prognoses. T2N1 NPC might have a greater risk of distant metastasis and poorer survival. Due to a lack of detailed data of individual patients, a subgroup analysis of stage II NPC was not performed. Hence, the role of adding concurrent chemotherapy to IMRT for T2 N1 patients requires further research. Several phases II-III trials (NCT02610010, NCT02116231, and NCT02633202) to evaluate the role of CCRT for stage II NPC patients treated with IMRT are ongoing. The results of these trials might throw light on this issue. The National Comprehensive Cancer Network (NCCN) and European Society for Medical Oncology (ESMO) recommend chemoradiation for stage II patients and consider it as category I recommendation [11].

## 3. Chemoradiation

The current standard of care for loco-regionally advanced (stages III-IV) NPC is concurrent chemoradiation.

Intergroup 0099 study conducted by Al Saraaf et al. [12] was the first landmark trial to show benefit of CCRT over RT alone in advanced NPC and was the key turning point of CCRT era. This study compared CCRT followed by adjuvant chemotherapy versus RT alone for patients with stages III-IVB disease in nonendemic areas. 193 patients were registered on to the study out of which 147 patients were eligible for analysis. At a median follow-up of 2.7 years, the 3-year actuarial progression free survival (PFS) was 24% and 69% in the RT and CCRT arm, respectively (p<0.001). The 3-year OS was 78% versus 47% in favor of CCRT arm. However, this study was not without its flaws. Around 22% of the patients in intergroup study had keratinizing type of tumors but more than 95% of the patients in endemic areas have nonkeratinizing or poorly differentiated carcinomas. The outcome of radiotherapy alone arm was much poorer than outcomes routinely achieved by RT arm of other trials. The proportions of patients who could complete the scheduled concurrent and adjuvant chemotherapy were only 63% and 55%, respectively. The RT technique used in the intergroup study was less aggressive than the techniques used in endemic areas, where parapharyngeal and intracavitary boost radiation were used in selected subsets of patients, thereby escalating total dose to the gross disease.

Wee et al. [13] tried to confirm the findings and applicability of the intergroup study in endemic areas. In this trial 221 patients were randomly assigned to receive RT alone (110) or CCRT (111). Patients in both arms received 70 Gy in 7 weeks using standard RT portals and techniques. Patients on CCRT received concurrent cisplatin-25 mg/m2 on days 1 to 4 on weeks 1, 4, and 7 of RT and adjuvant cisplatin (20 mg/m2 on days 1 to 4) and fluorouracil (1,000 mg/m2 on days 1 to 4) every 4 weeks (weeks 11, 15, and 19) for three cycles after completion of RT. The compliance to CCRT arm was reasonable with 71% of patients receiving the planned three cycles of concurrent chemotherapy during RT and 57% of patients completing all three cycles of adjuvant chemotherapy. The 2- and 3-year DFS rates were 57% and 75% and 53% and 72% for RT alone and CCRT patients, respectively. Thus, patients who were randomly assigned to receive CCRT had a lower risk of relapse. The 2- and 3-year survival rates were 78% and 85% and 65% and 80% for RT alone and CCRT, respectively. The distant metastasis was reduced by 17% in the CCRT arm as compared to RT arm.

To confirm the benefit of concurrent-adjuvant chemotherapy Hong Kong group [14] segregated stages III and IV NPC patients into two groups. Those with T1-4 N2-3 disease, accrued into the NPC-9901 trial, were irradiated with conventional fractionation and randomized to chemotherapy; those with T3-4 N0-1 disease, accrued into the NPC-9902 trial [15], were further randomly allocated to radiotherapy with conventional versus accelerated fractionation.

The NPC-9901 trial [14] on patients with T1-4N2-3M0 disease was designed to confirm the therapeutic benefit achieved by intergroup schedule. The failure-free survival (FFS) was significantly improved in the CCRT arm as compared to RT arm (At 3years 72% versus 62%; p=0.027). It was mostly as a result of an improvement in loco-regional control (92% versus 82%; p=0.005). However, distant control was not improved significantly and overall survival was identical in both arms. None of the subset got any OS benefit. The CCRT arm also had higher grade IV toxicities (12% versus 1%) and significantly higher incidence of RT related mucositis (62% versus 48%; p=0.01). In this study 65% of the patients could complete all six cycles and the mean total dose of CDDP was 444mg/m2; hence it was not suboptimal. Half of the patients in this study were treated with conformal radiotherapy throughout in both arms and boost was delivered to the involved site which might explain the minimal differential gain in outcome with chemotherapy.

The updated results of NPC- 9901 [16] trial showed that adding chemotherapy along with radiation statistically significantly improved the 5-year failure-free rate (FFR) [CCRT versus RT; 67% versus 55% p=.014] and 5-year PFS [CCRT versus RT; 62% versus 53% p=0.035]. This result was attributed to statistically significant improvement in 5-year loco-regional control (88% versus 78%; p=0.005). However, there was no significant difference between the distant metastasis failure-free rates. The OS rates were almost identical in both groups during the first 3 years and then showed a trend of improvement in the CCRT arm (68% versus 64% at 5 years and 61% versus 54% at 8 years (p=0.22). The CCRT arm had significantly higher incidence of acute toxicities (CCRT versus RT; 83% versus 53% p<0.001). The CCRT group also had higher late toxicities during the first 3 years but gradually leveled out at 5 years (30% versus 24%; p=0.30). The major limitation was patients with keratinizing squamous cell carcinoma and those with minimal lymphatic disease were not included. Hence this data cannot be extrapolated to all locally advanced NPC.

NPC-9902 trial [15] compared the benefit achieved by CCRT and/or accelerated fractionation (AF) versus RT alone with conventional fractionation (CF) for patients with T3-4N0-1M0 NPC. Between 1999 and April 2004, 189 patients were randomly assigned. When compared with the CF arm, significant improvement in FFS was achieved by the AF+C arm (94% versus 70% at 3 years,* p *= 0.008). However, the corresponding comparison with the AF arm and the CF+C arm did not show significant differences. There was no significant difference in overall survival between any of the arms. Both CCRT arms had significant increase in acute toxicities (p<0.005) and the AF+C arm also contributed borderline increase in late toxicities (34% versus 14% at 3 years, p=0.05). The magnitude of benefit achieved by CF+C arms compared to CF arm was minimal (FFS of 74% versus 70% and OS of 87% versus 83%).

Hong Kong group provided us with a cautionary note regarding blindly following the intergroup study and showed a different result compared to intergroup or Singapore study but follow-up period in those studies was less (around 3 years).

Chen et al. [17] performed a prospective randomized trial to evaluate the efficacy of CCRT versus RT alone in locally advanced nasopharynx in endemic areas of China. Patients received weekly concurrent cisplatin (40 mg/m2) followed by adjuvant cisplatin 80 mg/m2 on day 1 and fluorouracil 800 mg/m2 on day 1-day 5 every 4 weeks for three cycles. The RT dose was 70 Gy in 7 weeks using standard RT portals and techniques. The CCRT arm experienced significantly more acute toxicities (62.6% versus 32%; p≤0.001). A total of 68% and 61% of patients could complete all cycles of concurrent followed by adjuvant chemotherapy. The 2-year OS rate, FFS rate, distant failure-free survival rate, and loco-regional failure-free survival rate for the CCRT and RT groups were 89.8% versus 79.7% (p=0.003), 84.6% versus 72.5% (p=0.001), 86.5% versus 78.7% (p=0.024), and 98% versus 91.9% (p=0.007), respectively. Hence, this trial demonstrated significant survival benefit in favor of CCRT arm.

Conflicting results from different Asian groups raised some questions regarding effect of adding chemotherapy to radiotherapy on OS and led Baujat et al. to conduct the landmark MAC-NPC meta-analysis [2]. Eight trials with 1753 patients were included in this meta-analysis. The median follow-up was 6 years. Addition of chemotherapy added absolute OS benefit of 6% at 5 years (62% versus 56%). There was significant heterogeneity among different trials regarding the timing of chemotherapy. The concomitant trials showed a better treatment impact than induction or adjuvant chemotherapy. Overall there was 10% reduction in event free survival (EFS) at 5 years (52% versus 42%). CCRT significantly lowered the risk of both loco-regional failure (p=0.003) and distant failure (p=0.001). When they analyzed the interactions between treatment effect and patient characteristics the only significant interaction was found between WHO histologic type and effect of chemotherapy. The impact of chemotherapy was more prominent in WHO type I than type II or III disease (p=0.003 for OS and p<0.0001 for EFS). After exclusion of patients with WHO type I disease, the overall results remained significant in favor of CCRT arm. The result of this meta-analysis could not be simply attributable to intergroup study because the EFS benefit for the whole group of trials and the OS benefit in the concurrent group contributed by the chemotherapy remained statistically significant even after the exclusion of INT-0099 trial.

Blanchard et al. [18] updated the MAC-NPC meta-analysis with inclusion of more recent trials and analyzed separately the benefit of concurrent with and without adjuvant chemotherapy as distinct groups. The addition of chemotherapy to radiotherapy significantly improved OS with absolute benefit of 6.3% at 5 years (p<0.0001). The interaction between treatment effect (benefit of chemotherapy) on OS and the timing of chemotherapy was significant (p=0.01) in favor of concomitant plus adjuvant chemotherapy (HR 0.65, 0.56–0.76) and concomitant without adjuvant chemotherapy (0.80,0.70–0.93) but not adjuvant chemotherapy alone (0.87, 0.68–1.12) or induction chemotherapy alone (0.96,0.80–1.16). The benefit of the addition of chemotherapy was consistent for all endpoints analyzed (all p<0.0001): PFS (HR 0.75, 95% CI 0.69–0.81), loco-regional control (0.73, 0.64–0.83), distant control (0.67, 0.59–0.75), and cancer mortality (0.76, 0.69–0.84).

Zhang et al. [19] performed a meta-analysis of CCRT versus RT alone including studies conducted in endemic areas. There was OS advantage in two, three, and five years in favor of CCRT arm. In addition CCRT was associated with improved loco-regional and distant control. However, relative benefit of CTRT in endemic areas may not be as much as benefits observed in previous meta-analysis.

Most of the patients in the above-mentioned trials were treated with 2D or 3D conformal radiotherapy with IMRT in very limited cases. There is no published data from randomized control trial to address the role of CCRT with IMRT versus IMRT alone for locally advanced NPC.

Whether weekly cisplatin or 3 weekly cisplatin should be given with radiation was addressed in a phase 3 multicentre randomized controlled trial by Liang et al. [20] in which patients with stages II-IVB NPC were randomly assigned to receive either cisplatin 100 mg/m^2^ every 3 weeks for 2 cycles or cisplatin 40 mg/m^2^ weekly up to 6 cycles concurrently with IMRT. After a median follow-up of 17.5 months (range 1.6-64.1), estimated 2-year failure-free survival rate was 92% (95% CI 87.7-96.3) in weekly arm and 88.3% (95% CI 83.2-93.4) in three weekly arm (HR 1.056, 95% CI 0.58-1.92). Grade 3 or 4 toxicities were similar between two arms, but leucopenia and thrombocytopenia were significantly higher in weekly arm compared with three-week arm (24.8% versus 15.9%, P = 0.015 and 5.2% versus 1.1%, P = 0.01), respectively. This trial concluded that weekly regimen of cisplatin as CCRT shows similar treatment efficacy but increased toxic effect of leucopenia and thrombocytopenia compared with 3-week schedule in locally advanced NPC.

## 4. Whether Adding Adjuvant Chemotherapy to Concurrent Chemoradiation Is of Benefit?

The major goal of adjuvant chemotherapy was to reduce the subsequent occurrence of distant metastasis. Poor compliance with adjuvant chemotherapy limits its broader application. One major question regarding the design of intergroup 0099 study is the contribution of the adjuvant chemotherapy and its compliance. Only 55% patients of this study could complete the adjuvant phase. In Singapore study also only 57% of patients could complete the scheduled adjuvant treatment. Hong Kong group showed no benefit of adding adjuvant chemotherapy. Of the eight trials included in MAC-NPC meta-analysis only five had both concurrent and adjuvant component. In MAC-NPC update, out of 19 trials only six comparisons included concomitant plus adjuvant chemotherapy which made it difficult to identify the contribution of adjuvant chemotherapy.

Chen et al. [21] conducted a phase III multicentric randomized control trial to explore the effect of addition of adjuvant chemotherapy to standard CCRT in locally advanced NPC. At a median follow-up of 37.8 months the 2-year FFS was 86% in the CCRT plus adjuvant chemotherapy group as compared to 84% in CCRT only group. There were no significant differences in OS, distant metastasis failure-free survival, or loco-regional failure-free survival. Not a single subset of patients got any benefit from adding adjuvant chemotherapy. Compliance to three cycles of adjuvant chemotherapy was around 63% almost similar to other trials. The results need to be interpreted with caution because 20% of the patients discontinued the trial after starting adjuvant chemotherapy, 49% had dose reduction, and 69% experienced delay in treatment. This trial was not designed as a noninferiority trial against the standard intergroup 0099 trial; therefore negative results are difficult to interpret. Update of this trial reported in 2017 failed to demonstrate significant survival benefit for adjuvant cisplatin and fluorouracil chemotherapy after CCRT in loco-regionally advanced NPC after a median follow-up of 68.4 months (5-year FFS rate was 75% in the CCRT plus adjuvant chemotherapy group and 71% in the CCRT only group, HR 0.88, 95% confidence interval 0.64–1.22; p = 0.45) and addition of adjuvant cisplatin and fluorouracil did not significantly increase late toxicities [22].

CCRT plus adjuvant chemotherapy (AC) is associated with considerable toxicity and poor tolerance. Optimizing the AC regimen (for example, using oral Tegafur to replace 5-FU injection) might help in decreasing toxicity and enhancing the therapeutic efficacy. Trials are undergoing adopting this concept.

The use of biomarkers may guide us for tailoring treatment for a particular subset of patients who have a higher chance of distant metastasis and will benefit from adjuvant chemotherapy. Before treatment and after RT, EBV DNA levels are already correlated with survival and patient outcome [23, 24]. Chan et al. [25] found that relative risk of recurrence increased 11.9 times in patients with persistently elevated EBV DNA levels at 6-8 weeks after radiotherapy compared to patients without elevated EBV DNA levels.

The NPC 0502 trial [26] in Hong Kong addressed the issue whether patients with a detectable level of plasma EBV DNA at 6 weeks following CCRT should be given adjuvant chemotherapy. Only those patients with a detectable level of plasma EBV DNA after completing CCRT were randomized to undergo observation or six cycles of adjuvant cisplatin and gemcitabine. After median follow-up of 6.5 years (yr), the 5-year survival outcomes were similar between the two groups (RFS was 58.2 versus 57.3% and OS was 66.2% versus 67.6%)

Recently Hui et al. [27] prospectively validate plasma EBV DNA as the most significant prognostic biomarker in NPC that can be used to select high-risk patients for adjuvant therapy. The ERCC1 C118T genotype may help to identify a favorable subgroup (approximately 7%) of plasma EBV-negative patients with NPC who have an excellent prognosis and can be spared the toxicities of further therapy.

NRG HN 001 –NPC [28] is a phase II / III trial that is ongoing based on EBVDNA for loco-regionally advanced nonmetastatic NPC. All patients will first undergo standard CCRT. At the completion of CCRT, if there is no detectable plasma EBV DNA, then patients are randomized to either standard adjuvant cisplatin and fluorouracil chemotherapy or observation and if there is still detectable levels of plasma EBV DNA then patients will be randomized to standard cisplatin and fluorouracil chemotherapy versus gemcitabine and paclitaxel.

Although CCRT plus adjuvant chemotherapy (AC) has considerable toxicity, poor tolerance, and limited benefit, it is still recommended by NCCN guideline for loco-regionally advanced NPC.

## 5. Induction Chemotherapy Followed by CCRT

With the use of IMRT coupled with adoption of CCRT the loco-regional control has been improved and distant metastasis came out to be the predominant mode of treatment failure. When systemic agents were added in the adjuvant setting to reduce distant metastasis, the compliance to adjuvant chemotherapy was very poor as already shown in different trials. Hence induction chemotherapy followed by local treatment in the form of radiation or CCRT seems to be a logical strategy. The rationale behind the use of induction chemotherapy is based on two main clinical hypotheses: (1) the disease shrinkage and the subsequently RT volume reduction can allow more effective and less toxic RT; (2) multiple-agents up-front chemotherapy can influence distant metastases and OS.

The efficacy of induction chemotherapy followed by CCRT in locally advanced NPC is controversial and has shown some conflicting results.

Various phase 2 trials by Rischin et al. [29] and Hui et al. [30] suggested that induction chemotherapy followed by CCRT is a highly feasible approach with manageable toxicity profile and it also provided positive impact on survival.

Hellenic cooperative oncology group in a phase II study randomized 141 patients either to three cycles of induction chemotherapy with cisplatin, epirubicin, and paclitaxel (CEP) every 3 weeks followed by definitive RT (70 Gy) and concomitant weekly infusion of cisplatin (72 patients) or to standard CCRT regimen alone (69 patients). Overall and complete response rates were very similar in the two arms and so were 3-year PFS and OS rates. Grade III or IV toxic effects from induction chemotherapy were infrequent, apart from alopecia [31].

Tan et al. [32] conducted a randomized phases 2-3 trial comparing three cycles of induction gemcitabine, carboplatin, and paclitaxel chemotherapy followed by CCRT in stages III-IVB NPC and reported no difference in OS or PFS or distant metastasis failure-free survival between the two groups.

Su-Mei Cao et al. [33] conducted a randomized phase 3 trial to evaluate the feasibility and efficacy of induction chemotherapy with cisplatin (80 mg/m^2^ d1) and fluorouracil (800 mg/m^2^ iv d1–5) every 3 weeks for two cycles (PF Regimen) as induction followed by CCRT versus CCRT alone in loco-regionally advanced NPC. Both arms were treated with 80 mg/m^2^ cisplatin every 3 weeks concurrently with radiotherapy. There was higher 3-year DFS rate (82.0%, 95% CI = 0.77–0.87) in the induction followed by CCRT arm compared to CCRT alone (74.1%, 95% CI = 0.68–0.80, P = 0.028). The 3-year DMFS rate was 86.0% for the induction arm versus 82.0% for the control arm, with marginal statistical significance (P = 0.056). However, there were no statistically significant differences in OS or loco-regional relapse-free survival (LRRFS) rates between two arms (OS: 88.2% versus 88.5%, P = 0.815; LRRFS: 94.3% versus 90.8%, P = 0.430).

Sun et al. from China recently published the results of phase III study, where 241 patients were assigned to induction chemotherapy with three cycles of intravenous docetaxel (60 mg/m^2^ on day 1), cisplatin (60 mg/m^2^ on day 1), and continuous intravenous fluorouracil (600 mg/m^2^ per day from day 1 to day 5) every 3 weeks (TPF regimen) followed by CCRT arm and 239 to CCRT alone in locally advanced NPC (stage III-IVB except T3-T4N0). After a median follow-up of 45 months, 3-year FFS was 80% in the induction followed by CCRT group and 72% in the CCRT group (p=0.034). The 3-year OS was 92% in the induction arm compared to 86% in the CCRT arm (p=0.029). The 3-year distant failure-free survival was 90% and 83% in the induction and CCRT arms, respectively (p=0.031). The loco-regional control was similar in both arms. Grade 3/4 hematological toxicity was higher in the induction chemotherapy arm (neutropenia 42% versus 7%) and leucopenia (41% versus 17%). They have used TPF regimen which is already proven superior to PF regime. T3-T4N0 patients were excluded who have quite a low risk of distant metastasis which might have enhanced the power of this trial to show survival advantage. The dose of TPF was 20% lower than that of conventional regimen. During CCRT only 30% of the patients in the induction plus CCRT group and 56% patients in the CCRT alone group completed three cycles of concurrent cisplatin. This study does not include any prognostic biomarkers such as plasma EBV DNA load. They have excluded patients of 60 years or older. Follow-up period was also short (3 years) [34].

GEORTC trial [35] randomized 83 patients with locally advanced NPC to induction TPF plus concomitant cisplatin-RT or concomitant cisplatin-RT alone. The TPF regimen consisted of three cycles of docetaxel 75 mg/m^2^ day 1; cisplatin 75 mg/m^2^ day 1; 5FU 750 mg/m^2^/day days 1–5. RT consisted of 70 Gy in 7 weeks plus concomitant cisplatin 40 mg/m^2^ weekly. After a median follow-up of 43.1 months, the 3-year PFS rate was 73.9% in the TPF arm versus 57.2% in the CCRT alone arm (HR = 0.44; 95% CI: 0.20–0.97, *P* = 0.042]. Similarly the 3-year OS was 86.3% in the TPF arm versus 68.9% in the CCRT alone arm (HR = 0.40; 95% CI: 0.15–1.04, *P* = 0.05). The rate of grades 3–4 toxicity and the compliance during CCRT were not different between both arms.

A meta-analysis by Tan et al. [36] looked into the effects of addition of IC to CCRT versus CCRT alone on OS, PFS, DMFS, and adverse events (AE) in LA-NPC. Six RCTs and five observational studies including 2802 patients were included in the analysis. This meta-analysis showed that IC improved PFS (HR 0.69, 95% CI 0.57–0.84, *P* = 0.0003, *I*^2^ = 0%) and OS (HR 0.77, 95% CI 0.60–0.98, *P* = 0.03, *I*^2^ = 0%) significantly and was associated with more frequent AE.

The Hong Kong group [37] initiated a multicentre randomized controlled trial (NPC-0501) to evaluate three promising strategies (i) to change the chemotherapy strategies from concurrent-adjuvant to induction-concurrent, (ii) replacement of PF regimen with capecitabine, and (iii) reevaluating the potential benefit by changing conventional fractionation to accelerated fractionation. Preliminary results indicated that changing the sequence as demonstrated in comparison between induction PF and adjuvant PF did not show any significant difference in efficacy unadjusted comparisons of induction cisplatin and capecitabine (PX) versus adjuvant PF indicated a favorable trend in PFS for the conventional fractionation arm (p=0.045). Induction PX had lesser toxicities as compared to induction PF. Changing the fractionation from conventional to accelerated did not achieve any benefit but incurred higher toxicities (acute mucositis and dehydration).

The addition of IC to CCRT for LA-NPC can be considered as one of the standard treatment options for LA-NPC.

The role of immunotherapy in locally advanced nasopharyngeal cancer is not well-defined, though it was found beneficial in other head and neck sites.

## 6. Conclusion

The treatment of stage I nasopharyngeal carcinoma is RT alone. Chemoradiation is the standard of care for stage II and locally advanced nasopharyngeal ca. Among the different chemotherapy and radiation sequences concurrent chemoradiotherapy showed maximum benefit. Considerable toxicity, poor tolerance, and doubtful or limited benefit of adjuvant chemotherapy have made it less important approach. Induction chemotherapy approach is appealing in locally advanced Nasopharyngeal cancer. Identifying selected subset of patients for adjuvant chemotherapy based on postradiotherapy EBV DNA levels is a reasonable strategy to combat micrometastasis in the future.
